# Time-dependent efficacy of combination of silver-containing hydroxyapatite coating and vancomycin on methicillin-resistant *Staphylococcus aureus* biofilm formation in vitro

**DOI:** 10.1186/s13104-021-05499-7

**Published:** 2021-03-02

**Authors:** Akira Hashimoto, Hiroshi Miyamoto, Sakumo Kii, Tomoki Kobatake, Takeo Shobuike, Iwao Noda, Motoki Sonohata, Masaaki Mawatari

**Affiliations:** 1 Department of Orthopaedic Surgery, Faculty of Medicine, Saga University, Nabeshima 5-1-1, Saga, 849-8501 Japan; 2 Department of Pathology and Microbiology, Faculty of Medicine, Saga University, Nabeshima 5-1-1, Saga, 849-8501 Japan; 3 Research Section, Medical Division, KYOCERA Corporation, 800 Ichimiyake, Yasu City, Shiga 530-2362 Japan

**Keywords:** Biofilm, Hydroxyapatite, MRSA, Silver, Vancomycin

## Abstract

**Objective:**

We developed a silver-containing hydroxyapatite (Ag-HA) coating to prevent periprosthetic joint infection (PJI). Methicillin-resistant *Staphylococcus aureus* (MRSA) is the main PJI-causing bacteria. Previously, we had reported the combined effect of Ag-HA coating and vancomycin (VCM) on MRSA biofilm formation 24 h after MRSA inoculation. In this study, we investigated the time-dependent efficacy of Ag-HA coating and VCM on MRSA biofilm formation on Ti discs in vitro by three-dimensional confocal laser scanning microscopic analysis.

**Results:**

For the Ti VCM and HA VCM groups, the total biofilm volumes per area at 96 h after MRSA inoculation were significantly larger than those at 48 h after MRSA inoculation, respectively (p < 0.001). In contrast, for the Ag-HA VCM group, the total biofilm volume per area at 96 h was significantly smaller than that at 48 h (*p* < 0.0001). Moreover, 96 h after MRSA inoculation, the total biofilm volume per area of the Ag-HA VCM groups was significantly smaller than those of the Ti VCM and HA VCM groups (*p* < 0.0001). Thus, the combination of Ag-HA and VCM might be useful for the prevention of MRSA-associated PJI.

**Supplementary Information:**

The online version contains supplementary material available at 10.1186/s13104-021-05499-7.

## Introduction

Implantable medical device-related infections are caused by bacterial biofilm formation on these devices and are difficult to treat because of their resistance to antibiotics and immune cells [1]. Acute periprosthetic joint infection (PJI) is a devastating complication of total hip arthroplasty (THA) [2]. Introducing antibacterial coatings, developing anti-adhesion surfaces, and vaccination can be effective strategies for preventing device-associated infections [3]. Ag is a well-known antibacterial agent with a broader activity spectrum and lower bacterial resistance than antibiotics [4, 5]. Therefore, Ag-coated megaprostheses are used in orthopedic surgery [6]. However, inserting an Ag-coated prosthesis into the bone marrow is rather difficult as Ag is toxic to osteoblasts, suppresses ossification, and causes prosthesis loosening [7]. Meanwhile, hydroxyapatite (HA) accelerates early bone ingrowth and improves osteoconductivity [8]. Hence, we developed a silver-containing hydroxyapatite (Ag-HA) coating that effectively inhibits bacterial adhesion, enhances osteoconductivity, and is biomedically safe; it is deposited on Ti discs via thermal spraying [9–12].

The various mechanisms through which bacteria achieve antibiotic resistance include target-side mutation, antibiotic inactivation, and reduction of cytoplasmic antibiotic concentration [13, 14]. To overcome the infections related to drug-resistant bacteria, recent studies have proposed using hybrid antibiotics (combinations of antibiotics with either another antibiotic or with an adjuvant) [13]. PJI after THA is mainly caused by methicillin-resistant *Staphylococcus aureus* (MRSA) [15]. Earlier, we reported the combined effect of the Ag-HA coating and vancomycin (VCM) on MRSA biofilm formation 24 h after MRSA inoculation [16]. In this study, we investigated the time-dependent efficacy of the Ag-HA coating and VCM on MRSA biofilm formation in vitro.

## Main text

### Materials and methods

#### Ag-HA coating

Ag-HA was coated on one side of pure Ti discs (14 mm wide, 1 mm thick; Kobe Steel, Kobe, Japan) according to a previously reported method [16]. The Ag-HA coating technique is described in Additional file 1.

#### Preparation of bacterial culture

The MRSA strain used was UOEH6 (University of Occupational and Environmental Health Hospital, Fukuoka, Japan). It is a biofilm-producing strain and was isolated from the blood sample of a septic patient. The MRSA strain was cultured according to a previously reported method [16], which is described in Additional file 1.

#### Microbiological evaluation by bacterial count determination

Three types of discs were prepared: Ti, Ti with HA coating (HA), and Ti with 3.0% Ag-HA coating (Ag-HA). Microbiological evaluation was performed according to a previously reported protocol [16], which is described in Additional file 1. Twelve discs were used in each treatment group, namely Ti VCM, HA VCM, and Ag-HA VCM.

#### Three-dimensional confocal laser scanning microscopy (3D-CLSM) analysis

Four discs were used in each treatment group (Ti VCM, HA VCM, and Ag-HA VCM), and the MRSA cells were adhered onto the sample discs using the protocol used for microbiological evaluation. The total biofilm volume was determined by 3D-CLSM performed according to a previous study [16]. The method is described in Additional file 1.

### Statistical analyses

All numerical data are expressed as mean ± standard deviation. The normality distribution of continuous variables was evaluated by the Kolmogorov–Smirnov test. Live cell counts and the total biofilm volume per area for all the treatment groups were analyzed by the Steel–Dwass test. Live cell counts and the total biofilm volume per area at 48 h and 96 h for all the treatment groups were analyzed by the Wilcoxon signed-rank test. All analyses were performed using JMP Pro software (version 13.2.1; SAS Institute, Cary, NC, USA).

## Results

### Effect of treatments on bacterial survival

**Fig. 1:**
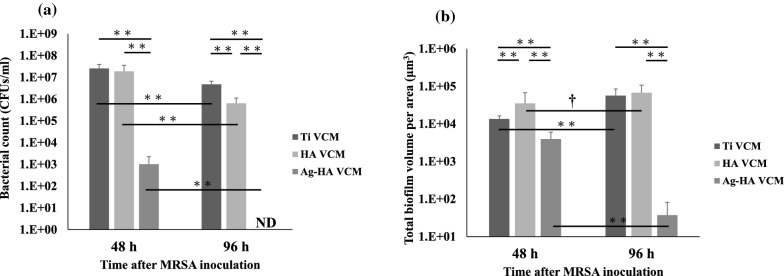
Effect of treatments on **a** bacterial survival and **b** biofilm formation. In **a**, VCM and Ag significantly reduced the bacterial cell count over time (n = 10 discs). In **b**, the total biofilm volume in the analyzed area in the Ag-HA VCM groups significantly decreased over time (n = 12 sections from 4 discs). Significant differences among three groups at 48 and 96 h and comparisons of groups at 48 and 96 h: †p < 0.01, **p < 0.001

As confirmed by plating, the discs were inoculated with (3.7 ± 1.5) × 10^8^ colony-forming units (CFU). The bacterial counts at 48 h for the Ti VCM, HA VCM, and Ag-HA VCM groups were (2.9 ± 0.9) × 10^7^, (1.9 ± 1.7) × 10^7^, (1.0 ± 1.2) × 10^3^ CFU/mL, respectively (Fig. 1a). At 96 h, the bacterial counts for the Ti VCM and HA VCM groups were (2.6 ± 1.4) × 10^7^ and (6.4 ± 4.8) × 10^5^ CFU/mL, respectively, while that for the Ag-HA VCM group could not be measured (Fig. 1a).

As shown in Fig. 1a, for all the groups, the bacterial counts at 96 h are significantly lower than those at 48 h, respectively (all *p* < 0.001). Particularly, the bacterial count of the treatment groups at 96 h decreased in the order of Ti VCM > HA VCM > Ag-HA VCM, with the bacterial count of the Ag-HA VCM group at 96 h being significantly lower than those of the Ti VCM and HA VCM groups at 96 h (all *p* < 0.001).

### Determination of total biofilm volume by CLSM

**Fig. 2:**
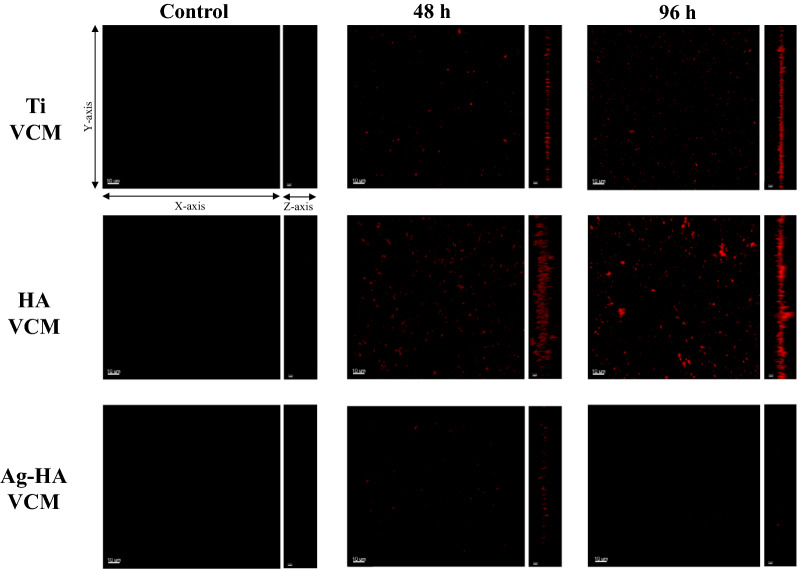
CLSM images of calcein red–orange-stained MRSA on discs. The bacterial growths from the following groups are shown: Ti, HA, and Ag-HA negative controls; Ti VCM, HA VCM, and Ag-HA VCM at 48 h; and Ti VCM, HA VCM, and Ag-HA VCM at 96 h. Scale bars for x and y-axes: 10 µm; scale bar for z-axis: 5 µm*.* The staining intensities of the biofilms are in the following order: Ag-HA VCM at 96 h < Ag-HA VCM at 48 h < Ti VCM at 48 h < Ti VCM at 96 h < HA VCM at 48 h < HA VCM at 96 h

As confirmed by plating, the discs were inoculated with (3.6 ± 1.7) × 10^8^ CFU bacterial cells. The total biofilm volume per area (Fig. 1b) was determined by analyzing the CLSM images (Fig. 2). For the Ti VCM, HA VCM, and Ag-HA VCM groups, the total biofilm volumes per area were (1.3 ± 0.3) × 10^4^, (3.5 ± 3.2) × 10^4^, and (3.9 ± 2.1) × 10^3^ µm^3^ at 48 h and (5.7 ± 2.8) × 10^4^, (6.8 ± 3.9) × 10^4^, and 37.2 ± 44.8 µm^3^ at 96 h, respectively (Fig. 1b).

As shown in Fig. 1b, for the Ti VCM and HA VCM groups, the total biofilm volumes per area at 96 h are significantly larger than those at 48 h, respectively (*p* < 0.001, *p* < 0.01). In contrast, for the Ag-HA VCM groups, the total biofilm volume per area at 96 h was significantly smaller than that at 48 h (*p* < 0.001). Particularly, the total biofilm volume per area of the treatment groups at 96 h decreased in the order of HA VCM > Ti VCM > Ag-HA VCM, with the total biofilm volume per area of the Ag-HA VCM group at 96 h being significantly smaller than those of the Ti VCM and HA VCM groups at 96 h (all *p* < 0.001).

## Discussion

Biofilm formation is a three-stage process involving bacterial adhesion, bacterial aggregation, and biofilm maturation [17]. Individual planktonic bacteria produce extracellular polymeric substances (EPS) after adhesion, which facilitate bacterium-to-bacterium adhesion. Thus, the biofilm thickness is directly proportional to EPS production. Moreover, EPS creates a diffusion barrier that prevents the uptake of antibiotics [18]. After biofilm maturation, the biofilm becomes more resistant to antibiotics [17]. Conversely, an early-stage biofilm is relatively unstable and less resistant to antibiotics than a mature biofilm [17]. As shown in Fig. 2, calcein red–orange stained the polysaccharide component of the biofilms, that is, bacteria and EPS, revealing the presence of early-stage biofilms in the Ag-HA VCM group.

New antibacterial methods are required to overcome the increasing drug resistance of bacteria [14]. Bacteria can reduce cytoplasmic antibiotic concentration by increasing active efflux though porins and decreasing permeability barriers [13, 14]. Efflux pump inhibitors play an important role in strengthening antibiotic effects on bacteria, and they are used with hybrid antibiotics [13, 14]. Siderophores and Aspergillomarasmine A are also used with hybrid antibiotics [13, 19, 20]. In addition, recent studies have proved the therapeutic potential of essential oils comprising plant-based compounds [21–24]. Essential oils show antibacterial and antibiofilm effects and could be used in synergistic therapy along with traditional antibiotics [21–24]. Although only VCM has no suppressive effect on MRSA biofilm formation, the combination of the Ag-HA coating and VCM showed powerful suppressive effects on MRSA biofilm formation in this study. Past studies have reported Ag as a potential efflux pump inhibitor [25, 26]. Therefore, the Ag-HA coating may also function as an efflux pump inhibitor.

Generally, VCM prophylaxis is not recommended for the prevention of surgical site infection (SSI) [27]. However, in MRSA carriers, VCM prophylaxis was found to be protective against MRSA-associated SSI [28]. However, in THA and total knee arthroplasty, VCM prophylaxis did not exhibit any substantial difference in the incidence of PJI compared with cefuroxime and fusidic acid prophylaxes [29]. Additionally, a recent study reported the presence of bacteria within the bone tissue in an osteomyelitis model, which may require extensive debridement for PJI treatment [30]. Therefore, implants with antibacterial coatings, which can be inserted into the bone marrow, are required to prevent PJI. In this study, VCM did not exhibit any suppressive effect on MRSA biofilm formation on materials without antibacterial coatings (Ti and HA). Contrarily, the combination of Ag-HA coating and VCM exhibited a powerful suppressive effect on MRSA biofilm formation. Hence, the combination of Ag-HA and VCM might be useful for the prevention of PJI in high-risk patients with MRSA-associated PJI.

## Conclusion

The combination of an Ag-HA coating and VCM exhibited a powerful suppressive effect on MRSA biofilm formation and can be a useful anti-infective approach for the prevention of MRSA-associated PJI.

## Limitations

This study was limited to an in vitro investigation. Therefore, the combined effect of the Ag-HA coating and VCM over time in an intramedullary implantation model (in vivo) should be investigated in the future.

## Supplementary Information

**Additional file 1.** Ag-HA coating method, preparation of bacterial culture, bacterial count determination, and 3D-CLSM analysis.

## Data Availability

The datasets used during this study are available from the corresponding author upon reasonable request.

## Abbreviations

Ag-HA: Silver-containing hydroxyapatite
3D-CLSM: Three-dimensional confocal laser scanning microscopy
HA: Hydroxyapatite
MRSA: Methicillin-resistant *Staphylococcus aureus*
PJI: Periprosthetic joint infection
SSI: Surgical site infection
THA: Total hip arthroplasty
Ti: Titanium
VCM: Vancomycin
