# Supplementary material for: Time-dependent efficacy of combination of silver-containing hydroxyapatite coating and vancomycin on methicillin-resistant Staphylococcus aureus biofilm formation in vitro
Source: BMC Res Notes. 2021 Mar 2;14:81. doi: 10.1186/s13104-021-05499-7 (PMC7927400; doi:10.1186/s13104-021-05499-7)
Supplement: Supplementary file 1 — Additional file 1. Ag-HA coating method, preparation of bacterial culture, bacterial count determination, and 3D-CLSM analysis. [file 13104_2021_5499_MOESM1_ESM.docx]

**Supplementary Information**

**Time-dependent efficacy of combination of silver-containing hydroxyapatite coating and vancomycin on methicillin-resistant Staphylococcus aureus biofilm formation *in vitro***

**Materials and methods**

**Fabrication of silver-containing hydroxyapatite (Ag-HA) coating**

Pure Ti discs (14 mm diameter, 1 mm thickness) were used as substrates for coating deposition. One side of the discs was sandblasted with a K5 sandblasting machine (TKX Corp., Osaka, Japan) with 180-grit aluminum oxide (Showa Denko K.K., Tokyo, Japan). The discs were then ultrasonically washed in ethanol for 3 min. Powdered silver oxide (Kanto Chemical, Tokyo, Japan) was added to powdered hydroxyapatite (HA; KYOCERA Corporation, Shiga, Japan) in a plastic bag and mixed for 5 min to prepare a 3% Ag-HA mixture. To coat the discs, HA powders with and without silver oxide were thermally sprayed onto the sandblasted surface using a flame-spraying system (Oerlikon Metco Japan Ltd., Tokyo, Japan) at a flame temperature of approximately 2700 °C. The spraying powder was carried into the flame by a dry air carrier gas during spraying, melted by the flame, and then sprayed onto the discs. The Ti discs were coated under normal atmospheric pressure. The physical and chemical properties of Ag-HA have been previously reported [1,2]. The discs were individually packaged and sterilized using a JS-8500 gamma sterilizer (MDS Nordion, Ontario, Canada).

**Preparation of bacterial culture**

The MRSA strain used was UOEH6 (University of Occupational and Environmental Health Hospital, Fukuoka, Japan). It is a biofilm-producing strain and was isolated from the blood sample of a septic patient. Bacteria were cultured overnight in tryptic soy broth (Eiken Chemical, Tokyo, Japan) at 37 °C and then centrifuged. The pelleted cells were rinsed and re-suspended in heat-inactivated 100% fetal bovine serum (Thermo Fisher Scientific, Wilmington, DE, USA) to obtain a cell concentration of approximately 10^8^ CFU/mL. Immediately after inoculation, serial dilutions of the residual suspension were prepared, plated on agar plates, and incubated for 48 h at 37 °C. The colonies were counted, and the CFU/mL was determined.

**Microbiological evaluation by bacterial count determination**

Three types of discs were prepared: Ti, Ti with HA coating (HA), and Ti with 3.0% Ag-HA coating (Ag-HA). The inverted lid of a 60-mm-diameter dish was placed in the center of a 90-mm-diameter dish. Three or four discs of the same type were aseptically placed on the inverted lid of the 60-mm-diameter dish, and 10 µL of MRSA suspension was inoculated onto each disc. Then, 10 µL of 20 µg*/*mL VCM (Kobayashi Kakou Corp., Fukui, Japan) was immediately added to each disc. To prevent drying, sterile phosphate-buffered saline (PBS) was poured between the 90- and 60-mm-diameter dishes, and the 90-mm-diameter dish was covered with a parafilm. The discs were incubated for 48 h or 96 h at 37 °C, agitated with a vortex mixer in 10 mL sterile PBS, ultrasonically irrigated for 5 min, and agitated again with the vortex mixer. Finally, serial dilutions (10-fold series) of the irrigated PBS solutions were plated on agar plates and incubated for 48 h at 37 °C. The colonies were counted, and the CFU per disc was determined. Ten discs were used in each treatment group, namely Ti VCM, HA VCM, and Ag-HA VCM.

**Three-dimensional confocal laser scanning microscopy (3D-CLSM)**

Four discs were used in each treatment group (Ti VCM, HA VCM, and Ag-HA VCM). MRSA cells were adhered onto the sample discs by the protocol used for microbiological evaluation. All discs were rinsed twice with 500 µL of sterile PBS to remove non-adherent cells, stained with calcein red-orange biofilm stain (FilmTracer calcein red-orange biofilm stain, Thermo Fisher Scientific) for 1 h, and then washed twice with 500 µL of sterile PBS. The stained biofilms were observed under a confocal laser scanning microscope with a 20× air objective lens (LSM880, Carl Zeiss AG, Jena, Germany) after excitation at 543 nm with a He–Ne laser. An area of 0.208 µm (x-axis) × 0.208 µm (y-axis) was screened at 1 µm intervals along the z-axis (z-stack) in the red emission spectrum (548 to 640 nm) at a resolution of 1024 × 1024 pixels with a zoom factor of 2.0 at a scan time of 8.8 s. The pinhole was adjusted to 32 µm. The total biofilm volume per area was determined using IMARIS image analysis software (Carl Zeiss AG). Because the biofilm dimensions varied, three sections of each disc were randomly scanned for quantitative analysis according to the method described in literature [3,4].

**References**

# Noda I, Miyaji F, Ando Y, Miyamoto H, Shimazaki T, Yonekura Y, et al. Development of novel thermal sprayed antibacterial coating and evaluation of release properties of silver ions. J Biomed Mater Res B Appl Biomater. 2009;89(2):456–65.

# Shimazaki T, Miyamoto H, Ando Y, Noda I, Yonekura Y, Kawano S, et al. In vivo antibacterial and silver-releasing properties of novel thermal sprayed silver-containing hydroxyapatite coating. J Biomed Mater Res B Appl Biomater. 2010;92(2):386–9.

# Hashimoto A, Miyamoto H, Kobatake T, Nakashima T, Shobuike T, Ueno M, et al. The combination of silver-containing hydroxyapatite coating and vancomycin has a synergistic antibacterial effect on methicillin-resistant *Staphylococcus aureus* biofilm formation. Bone Joint Res. 2020;9(5):211–8.

# [Klinger-Strobel M](https://www.ncbi.nlm.nih.gov/pubmed/?term=Klinger-Strobel%20M%5BAuthor%5D&cauthor=true&cauthor_uid=28267594), [Stein C](https://www.ncbi.nlm.nih.gov/pubmed/?term=Stein%20C%5BAuthor%5D&cauthor=true&cauthor_uid=28267594), [Forstner C](https://www.ncbi.nlm.nih.gov/pubmed/?term=Forstner%20C%5BAuthor%5D&cauthor=true&cauthor_uid=28267594), Makarewicz O, Pletz MW. Effects of colistin on biofilm matrices of *Escherichia coli* and *Staphylococcus aureus*. [Int J Antimicrob Agents](https://www.ncbi.nlm.nih.gov/pubmed/?term=Effects+of+colistin+on+bio%EF%AC%81lm+matrices+of+Escherichia+coli+and+Staphylococcus+aureus). 2017;49(4):472–9.
